# Nanozyme-Driven Signal Amplification in Cancer Biosensing: Innovations Toward Precision and Point-of-Care Oncology

**DOI:** 10.3390/mi17050541

**Published:** 2026-04-28

**Authors:** Victor Akpe, Ian E. Cock

**Affiliations:** School of Environment and Science, Griffith University, Nathan Campus, Brisbane, QLD 4111, Australia; v.akpe@scholarflow.co.site

**Keywords:** nanozyme-enabled biosensors, cancer biomarker detection, amplification-free assays, microfluidic integration, point-of-care diagnostics, translational oncology

## Abstract

This review evaluates recent progress in nanozyme-based biosensors for detecting circulating tumour cells, nucleic acids, and protein biomarkers, with particular attention to how peroxidase-, oxidase-, and catalase-like reactions enhance signal generation across electrochemical, optical, and microfluidic platforms. The roles of iron oxide–gold composites, silica nanostructures, quantum dots, and hybrid nanomaterials in improving analytical performance, enabling multiplexed detection, and facilitating assay miniaturization are critically assessed. Advances such as amplification-free detection approaches, smartphone-compatible point-of-care systems, and AI-assisted data analysis are discussed in relation to their translational potential. Key barriers, including regulatory requirements, reproducibility concerns, and manufacturing scalability, are also evaluated. By integrating mechanistic understanding with practical considerations for clinical deployment, this review outlines how next-generation nanozyme-based biosensors may strengthen early cancer detection, real-time monitoring, and precision oncology.

## 1. Introduction

Cancer remains a major global health burden, causing nearly 10 million deaths each year across both high-income and low-income regions [1]. Although imaging, histopathology, and molecular diagnostics have improved substantially, many cancers are still identified at advanced stages, when treatment options are limited and prognosis remains poor [2,3]. Improving early detection and enabling continuous monitoring are therefore essential for improving survival outcomes. However, current diagnostic approaches often remain invasive, costly, and insufficiently sensitive [4,5,6]. These limitations have accelerated efforts to develop technologies capable of delivering rapid, accurate, and minimally invasive cancer diagnostics.

Biosensors offer a promising pathway to address these challenges by enabling real-time detection of cancer-associated biomarkers with high analytical sensitivity and specificity [7]. By integrating biological recognition elements with diverse transduction mechanisms, biosensor platforms can quantitatively detect proteins, nucleic acids, and whole cells in complex biological samples [8,9]. In oncology, these capabilities have been applied to the detection of circulating tumour cells (CTCs), circulating tumour DNA (ctDNA), and exosomal markers, providing clinically relevant information on tumour burden and therapeutic response [10]. Continued miniaturization of biosensor systems further strengthens the clinical utility of point-of-care testing (POC) and expands access to diagnostic tools beyond centralized laboratories [11].

This review specifically evaluates nanozyme-enabled biosensors for detecting circulating tumour cells, nucleic acids, and protein biomarkers, with emphasis on how catalytic mechanisms drive signal amplification across electrochemical, optical, and microfluidic platforms. The section also outlines the translational relevance of these systems, including their potential for point-of-care deployment and integration with emerging digital diagnostics.

Nanomaterials have played a central role in advancing biosensor performance by enhancing signal amplification, improving surface functionalization, and enabling multiplexed detection [12]. Silica nanostructures, quantum dots, and hybrid composites offer tunable optical and electronic properties that can be tailored for specific biomarker targets [13,14]. These nanomaterial-based systems routinely achieve sensitivities surpassing those of conventional assays, including femtomolar detection limits and single-cell resolution in CTC analyses [15]. Such progress points to the growing influence of nanotechnology in cancer diagnostics.

As a distinct class of nanomaterials, nanozymes mimic the catalytic activity of natural enzymes whilst offering robustness, tunability, and cost effectiveness [16]. Unlike biological enzymes, which are often unstable under harsh conditions, nanozymes maintain catalytic activity across diverse environments, including variations in pH and temperature [17]. Nanozymes with peroxidase-like, oxidase-like, and catalase-like activities support diverse sensing modalities, from colorimetric assays to electrochemical detection [18]. The ability to engineer surface chemistry and particle morphology further enhances nanozyme catalytic efficiency and facilitates their integration into biosensor platforms [19].

Compared with natural enzymes, nanozymes offer several practical advantages: they are less expensive to manufacture, more stable during storage and operation, and readily engineered for multiplexed detection [20]. Catalytic properties can be precisely tuned through modifications in particle size, composition, and surface ligands, allowing control over biosensor performance [21]. Nanozymes also integrate effectively with optical, electrochemical, and microfluidic platforms, broadening their applicability across diverse diagnostic platforms [22,23]. Overall, these attributes position nanozymes as strong candidates for sensitive and reliable biomarker detection.

Recent progress in CTC research highlights the importance of integrating biosensors into cancer monitoring workflows to capture clinically meaningful signals from blood-based assays [24]. Previous studies have shown that Fe_3_O_4_–Au nanozymes can enhance catalytic amplification in cancer biomarker assays, supporting their integration into translational biosensor platforms [25]. Building on these foundational contributions, this review integrates emerging literature to provide a comprehensive evaluation of nanozyme-enabled biosensors in oncology. By positioning biosensor innovation within the broader landscape of global cancer diagnostics and the core principles of nanozyme science, we establish a framework for examining their translational potential from laboratory validation to clinical implementation and future applications.

## 2. Nanozyme Fundamentals

A central component of these advances is the emergence of nanozymes, which exhibit enzyme-like catalytic behaviour whilst offering enhanced robustness and design flexibility [26]. The combination of catalytic efficiency, stability, adaptability, and multifunctionality has expanded nanozyme relevance in biosensing applications [27]. These characteristics are particularly valuable in cancer diagnostics, where analytical sensitivity and reproducibility are essential [28].

Peroxidase-like catalysis in nanozymes is primarily governed by vacancy mediated adsorption of H_2_O_2_ and rapid electron shuttling between metal centres, enabling efficient generation of highly reactive ^•^OH radicals that drive strong signal amplification in biosensing assays [29]. Iron oxide–gold (Fe_3_O_4_–Au) nanozyme composites demonstrate a dual catalytic and bio-interfacing role, combining the magnetic responsiveness of Fe_3_O_4_ with the plasmonic, catalytic, and bioconjugation advantages of Au. This synergy enhances signal output in colorimetric and electrochemical platforms and provides a robust scaffold for biomarker binding conjugate applications [30,31]. Nanozyme-driven signal amplification therefore holds strong potential to improve assay sensitivity and reproducibility in translational oncology. Nanozyme performance is also shaped by intrinsic physicochemical features such as surface defect density, oxygen vacancy distribution, and electron transfer efficiency, all of which influence catalytic turnover and substrate affinity.

Another important class of nanozymes is the oxidase-like, which catalyse oxygen-dependent reactions without requiring hydrogen peroxide, enabling simplified assay cascades [32]. Cerium oxide and manganese oxide nanostructures fall into this class. For example, cerium oxide and manganese oxide nanostructures can oxidize substrates such as o-phenylenediamine and dopamine, generating clear and measurable colorimetric signals [33]. Oxidase-like activity is supported by the ability of nanozymes to activate dissolved molecular oxygen at defect-rich surface sites, enabling multi-step electron transfer processes that drive substrate oxidation in the absence of exogenous H_2_O_2_, an advantage that enhances practicality for point-of-care biosensors [34].

Catalase-like nanozymes constitute another major class, decomposing hydrogen peroxide into water and oxygen in a manner analogous to natural catalase enzymes [35]. This catalytic mode is particularly relevant for monitoring reactive oxygen species (ROS) in tumour microenvironments and for biosensors designed to assess oxidative stress [36]. Platinum-based and iron oxide nanozymes frequently exhibit catalase-like behaviour, supporting applications in cancer diagnostics and therapeutic monitoring [37]. Catalase-like activity is facilitated by high-energy active sites that promote rapid H_2_O_2_ decomposition, supported by favourable electron donor/acceptor interactions and reduced activation barriers at engineered surface defects.

**Figure 1:**
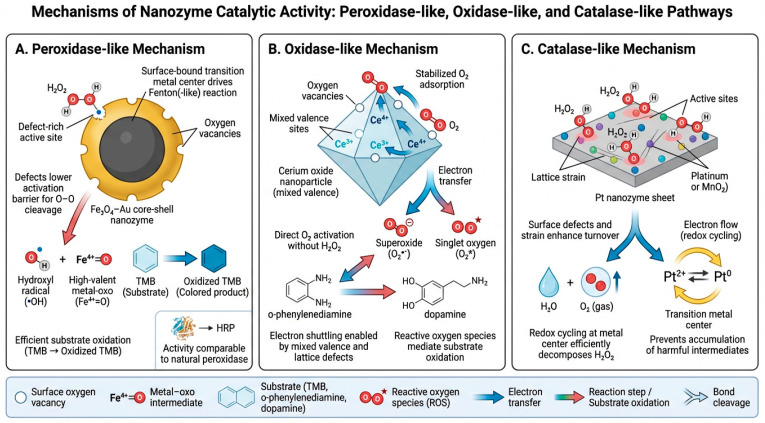
Mechanistic pathways of nanozyme catalytic activity across peroxidase-like, oxidase-like, and catalase-like modes. (**A**) Peroxidase-like mechanism (Fe_3_O_4_–Au nanozymes). Defect-rich Fe_3_O_4_–Au core–shell nanozymes catalyse Fenton-type reactions in the presence of H_2_O_2_. Surface-bound transition metal centres (Fe^2+^/Fe^3+^) and oxygen vacancy sites lower the activation barrier for O–O bond cleavage, generating highly reactive hydroxyl radicals (•OH) and high valent metal–oxo intermediates (Fe^4+^=O). These species oxidize chromogenic substrates such as TMB, producing a measurable colour change. Electron transfer pathways between Fe^2+^/Fe^3+^ and the Au shell accelerate redox cycling and enhance turnover. Symbols: oxygen vacancies (౦), metal–oxo intermediates (Fe^4+^=O), electron transfer arrows, and substrate-to-product conversion. Relevance to biosensing: this pathway highlights colorimetric and electrochemical amplification strategies, enabling ultrasensitive detection of cancer biomarkers through strong signal generation. (**B**) Oxidase-like mechanism (CeO_2_ nanozymes). Cerium oxide nanoparticles with mixed Ce^3+^/Ce^4+^ valence states activate dissolved O_2_ directly at defect-rich surfaces without requiring H_2_O_2_. Oxygen vacancies stabilize O_2_ adsorption and promote multi-step electron transfer, producing reactive oxygen species such as superoxide (O_2_•^−^) and singlet oxygen (^1^O_2_). These ROS oxidize substrates including o phenylenediamine and dopamine, generating quantifiable optical signals. Mixed valence cycling (Ce^3+^ ↔ Ce^4+^) supports continuous electron shuttling and sustained catalytic activity. Symbols: Ce^3+^/Ce^4+^ redox pairs, oxygen vacancy sites, ROS species (O_2_•^−^, ^1^O_2_), and substrate oxidation arrows. Relevance to biosensing: oxidase-like nanozymes eliminate the need for exogenous H_2_O_2_, simplifying assay design and improving compatibility with point-of-care platforms. (**C**) Catalase-like mechanism (Pt or MnO_2_ nanozymes). Platinum-based sheets and MnO_2_ nanosheets decompose H_2_O_2_ into H_2_O and O_2_ via redox cycling at high-energy active sites. Lattice strain and surface defects enhance electron flow between Pt^2+^/Pt^0^ (or Mn^4+^/Mn^2+^) states, preventing accumulation of harmful intermediates and enabling rapid turnover. The catalytic process produces molecular oxygen, which can be quantified directly or used to modulate downstream reactions. Symbols: active site redox pairs (Pt^2+^/Pt^0^), electron transfer arrows, O_2_ gas evolution, and defect-rich catalytic surfaces. Relevance to biosensing: catalase-like activity supports ROS-related biomarker detection, tumour microenvironment profiling, and oxygen-generation-based signal modulation in cancer diagnostics. Overall significance: across all three panels, the figure highlights how surface defects, oxygen vacancy density, lattice strain, and electron transfer pathways govern nanozyme catalytic behaviour. These mechanistic determinants directly influence signal amplification in nanozyme-enabled biosensors, enabling sensitive, robust, and tunable detection strategies for cancer biomarkers.

The catalytic performance of nanozymes is shaped by surface chemistry, electronic structure, and particle morphology [38]. As illustrated in Figure 1, nanozyme activity depends on electron transfer pathways influenced by transition metal ions, oxygen vacancies, and surface defects, all of which play central roles in driving catalytic reactions [39]. Furthermore, the mechanistic framework of Fe_3_O_4_–Au composites exemplify how synergistic interactions between the magnetic iron oxide core and the plasmonic gold shell accelerate catalytic kinetics, leading to higher turnover rates and enhanced analytical sensitivity [40]. A mechanistic understanding of these interactions is essential for the rational design and optimization of nanozyme-based biosensors.

Recognizing the diversity of nanozyme activities enables researchers to match catalytic behaviour with specific diagnostic requirements. For example, peroxidase-like activity is well suited for colorimetric assays, oxidase-like activity reduces reagent complexity, and catalase-like activity provides access to oxidative stress-related biomarkers [41]. Leveraging these catalytic modes allows nanozyme-enabled biosensors to achieve high sensitivity, strong reproducibility, and broad adaptability across a range of cancer diagnostic applications [42], Table 1 compiles the summary of nanozyme classes and mode of catalytic activity, along with some of their specific applications. Additionally, a consolidated overview of these nanozyme classes, including catalytic functions, detection modalities, analytical performance, and clinical readiness, is presented in Table 2, which expands on these distinctions to guide selection for biomarker-specific biosensor design.

## 3. Nanomaterials in Biosensor Platforms

Within this broader framework, silica nanomaterials play a central role in biosensor engineering due to their biocompatibility, high surface area, and tunable porosity, features that make them effective scaffolds for molecular recognition [56]. When immobilised with antibodies, aptamers, and enzymes, they provide stable recognition interfaces for cancer biomarker detection [57]. Mesoporous silica nanoparticles (MSNs) further support controlled probe loading and diffusion regulated release, improving assay reproducibility and sensitivity in complex biological samples [58]. Silica nanostructures exhibit enhanced biosensing performance through defect-mediated surface chemistry, where silanol group density, pore-wall charge distribution, and engineered surface defects modulate probe loading, molecular diffusion, and electron transfer efficiency. These mechanistic features directly influence binding kinetics, signal amplification, and assay reproducibility in silica-based biosensors [59].

**Figure 2:**
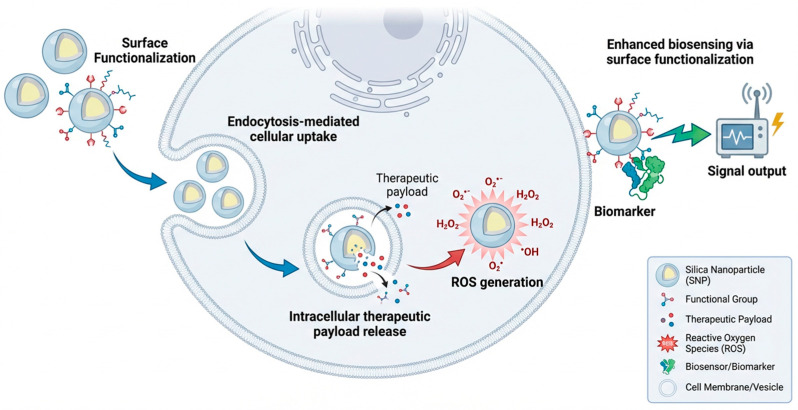
Silica nanostructures for nanomedicine. Schematic representation of silica nanoparticle-mediated mechanisms in nanomedicine. Cellular uptake occurs via endocytosis, enabling intracellular delivery. Silica nanostructures facilitate ROS generation and therapeutic payload release, whilst also enhancing biosensing capabilities through surface functionalization and signal transduction.

Figure 2 illustrates the multifunctional roles of silica nanostructures in nanomedicine, showing how surface-engineered silica nanoparticles (SNPs) participate in cellular uptake, intracellular delivery, and biosensing. Once internalized through endocytosis, SNPs can mediate stimuli-responsive release of therapeutic payloads and influence intracellular redox balance through interactions that modulate reactive oxygen species (ROS). The ROS enter cells through endocytosis, enabling controlled release of therapeutic payloads once internalized. In parallel, surface-functionalized silica nanostructures enhance biosensing performance by providing chemically tailored interfaces that stabilize biorecognition elements and improve signal transduction efficiency.

Another important class of materials for biosensor development is quantum dots (QDs), which are a unique set of semiconductor nanocrystals with distinctive photophysical properties. A striking feature of the functional application of QDs is that they have a broad range of fluorescence emission that is size-dependent and has a high quantum yield and strong resistance to photobleaching, making QDs particularly suitable for optical biosensing, where signal stability and tunability are essential [60,61]. The photophysical behaviour of QDs is governed by quantum confinement effects, surface trap states, and engineered core–shell interfaces, which collectively regulate emission wavelength, quantum yield, and photostability. These mechanistic determinants underpin their sensitivity in fluorescence-based biosensors, enabling precise modulation of signal output in complex biological environments.

Significant advancements in graphene quantum dot (GQD) composites for cancer diagnosis and biosensing applications have reported enhanced functionalisation when combined with various materials, such as gold (AuNPs), carbon nanotubes (CNTs), polyaniline (PANI), and zeolitic imidazolate framework-8 (ZIF-8), to create composites with improved sensitivity, selectivity, and stability [62]. Recent advances in quantum dot imaging have shown how atomic-level structural control can improve emission stability and spectral tunability [63]. Hybrid GQD composites leverage synergistic charge transfer pathways, defect-rich interfaces, and plasmon–exciton coupling to enhance fluorescence intensity, reduce non-radiative recombination, and improve target binding specificity. Atomic-level structural control further stabilizes exciton dynamics, enabling highly tunable and robust optical responses for biosensing.

Silica-coated QDs represent another important evolution in QD engineering, offering improved stability and biocompatibility for biomedical applications [64]. Silica shells enhance physicochemical robustness, reduce cytotoxicity, and convert hydrophobic QD surfaces into hydrophilic interfaces suitable for aqueous biological systems. These coatings preserve high photoluminescence efficiency, minimize photobleaching, and extend fluorescence lifetimes, making silica-coated QDs valuable for imaging and sensing applications. Silica shells modulate QD behaviour by passivating surface trap states, stabilizing exciton recombination pathways, and creating controlled dielectric environments that enhance fluorescence lifetime and emission stability. These mechanistic improvements directly translate into higher signal fidelity and reduced photodegradation in biosensing applications.

**Figure 3:**
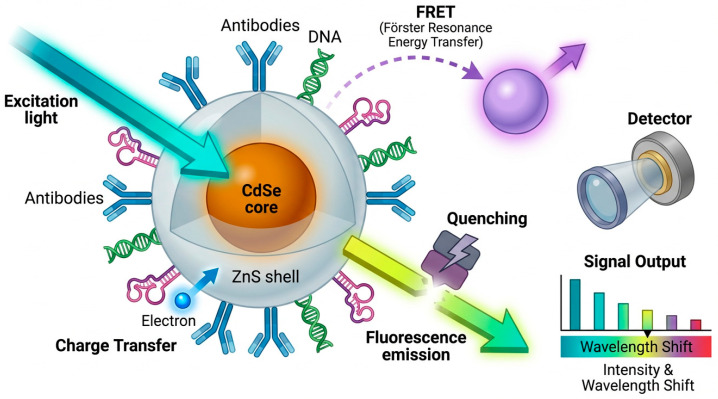
QD fluorescence modulation mechanism. Advanced schematic illustrating fluorescence modulation of core-shell QDs (CdSe/ZnS) via excitation and emission pathways. Surface functionalization with biomolecules (antibodies, DNA, aptamers) enables FRET, quenching, and charge transfer interactions. These mechanisms modulate the fluorescence signal, producing measurable changes in signal output such as intensity shifts and wavelength variations for biosensor applications.

The schematic in Figure 3 illustrates how excitation and emission pathways in QD systems are influenced by surface-bound biomolecules such as antibodies, DNA, and aptamers. These functional groups enable interactions including FRET, quenching, and charge transfer, each of which alters fluorescence output. Fluorescence modulation in QD systems is driven by nanoscale energy-transfer processes, including donor–acceptor distance-dependent FRET, surface-mediated charge-transfer quenching, and exciton–biomolecule coupling. These mechanistic interactions precisely tune emission intensity and spectral shifts, enabling highly selective biomarker detection. The resulting changes in signal intensity or wavelength form the basis for sensitive and selective detection in QD-based biosensors.

Altogether, the design of QD-based systems increasingly incorporates principles of self-assembly, in which nanoscale components autonomously organize into ordered structures through non-covalent interactions [65]. This strategy facilitates the creation of multifunctional architectures with enhanced optical, chemical, and biological performance. Self-assembly processes exploit electrostatic interactions, hydrogen bonding, and ligand-mediated coordination to organize QDs, silica matrices, and metallic nanoparticles into ordered architectures. These controlled nanoscale arrangements optimize energy transfer, probe accessibility, and multimodal signal integration in hybrid biosensor platforms.

A notable example of such hybrid engineering is the development of QD@MSN-EPI-Au-PEG-Apt nanoparticles for colorectal cancer theranostics [66]. These multimodal constructs combine QDs, MSNs, and Au nanoparticles to achieve targeted drug delivery, radiosensitization, and multimodal imaging. The platform incorporates epirubicin within the silica matrix, Au nanoparticles for enhanced radiotherapy response, PEGylation for improved circulation, and EpCAM aptamers for selective targeting of colorectal cancer cells. The QD@MSN–Au hybrid platform demonstrates how hierarchical nanoconstructs integrate optical, catalytic, and targeting mechanisms through coordinated energy transfer, enhanced radiative-decay modulation, and ligand-directed cellular uptake. These mechanistic synergies enable simultaneous imaging, drug delivery, and therapeutic enhancement.

In summary, advances in QD engineering, from intrinsic photophysical tuning to silica coating, controlled self-assembly, and hybrid nanoconstruct design, have substantially expanded the capabilities of nanomaterial-based biosensors. These innovations support ultrasensitive detection, multiplexed analysis, and device miniaturization, whilst enabling translational applications such as POC diagnostics and multimodal imaging [67,68]. Collectively, these engineering strategies refine exciton dynamics, surface-state energetics, and nanoscale charge-transfer pathways, enabling biosensor platforms with superior sensitivity, multiplexing capability, and translational robustness [69].

## 4. Cancer Biomarker Detection Using Nanozymes

Nanozyme-enabled biosensors have shown exceptional sensitivity in detecting protein biomarkers associated with cancer progression. Peroxidase-like iron oxide–gold (Fe_3_O_4_–Au) nanozymes catalyse colorimetric reactions that amplify signals from low-abundance proteins such as prostate-specific antigen (PSA) and carcinoembryonic antigen (CEA) [70]. Functionalization with antibodies or aptamers has enabled picomolar-level detection, surpassing the performance of conventional ELISA assays [71]. These developments demonstrate the potential of nanozymes to deliver rapid, cost-effective, and clinically relevant protein biomarker assays [72].

Furthermore, nanozymes have been applied to nucleic acid detection, including circulating tumour DNA (ctDNA) and microRNAs (miRNAs), which serve as key indicators of tumour burden and therapeutic response [73]. Catalytic amplification by Fe_3_O_4_–Au nanozymes enables sensitive detection of short nucleic acid sequences whilst reducing dependence on conventional amplification steps such as PCR, which are commonly but not universally used in nucleic acid-based diagnostic workflows [74]. By minimizing the need for these amplification procedures, nanozyme-enabled platforms lower assay complexity and shorten turnaround times, thereby improving suitability for POC applications [75]. When integrated with electrochemical biosensors, nanozyme-based systems can further discriminate single-nucleotide variants in cancer-associated genes, enhancing analytical specificity [76].

Moreso, nanozyme-based biosensors have also been adapted for whole cell detection, particularly circulating tumour cells (CTCs), which are critical indicators of metastasis and disease progression [77]. Magnetic Fe_3_O_4_–Au nanozymes enable both selective capture and catalytic amplification, supporting dynamic detection ranges from 10 to 10^5^ cells/mL [78]. Advances in cytosensor design, such as three-site recognition strategies using branched Pt–Au nanospheres and MnO_2_–GO–Au nanosheets have further improved selectivity and accuracy in CTC detection [79]. These innovations demonstrate the value of nanozyme-functionalized biosensors as robust platforms for translational oncology, bridging laboratory assay development with clinical monitoring of tumour dissemination.

Case studies in breast cancer biomarker detection further illustrate the translational potential of advanced biosensor platforms. The development of label-free multiplex electrochemical biosensors capable of simultaneously detecting MUC1, CA15-3, and HER2 has demonstrated improved sensitivity and reproducibility compared with conventional assays [80]. Multiplexed biosensing continues to accelerate clinical translation by enabling simultaneous quantification of multiple disease-relevant biomarkers within a single assay cascade. Recent studies have expanded this capability to include additional breast cancer markers such as HER2, CA15-3, and other clinically significant proteins [81]. Altogether, these applications show how nanozymes can be harnessed for protein, nucleic acid, and cell-based detection, positioning them as key components of next-generation cancer diagnostics [82].

## 5. Integration with Biosensor Technologies

Electrochemical biosensors have become widely used in oncology due to their high sensitivity, rapid response, and compatibility with miniaturized diagnostic platforms [83]. Incorporating nanozymes into these systems enhances electron transfer and catalytic amplification, enabling detection of cancer biomarkers at ultra-low concentrations [84]. Iron oxide-based nanozymes have been integrated into electrochemical interfaces to strengthen catalytic signal output, supporting highly sensitive detection of clinically relevant biomarkers, including those associated with breast cancer [85].

Optical biosensors, including fluorescence and colorimetric platforms also benefit from nanozyme-mediated catalysis, which amplifies optical signals and improves assay reproducibility [86]. QD-based optical platforms extend these capabilities by enabling multiplexed detection, allowing simultaneous quantification of multiple biomarkers within complex biological samples [87].

Microfluidic biosensors represent another major advancement in cancer diagnostics, offering precise fluid manipulation, sample miniaturization, and high-throughput analysis [88]. Incorporation of nanozymes into microfluidic channels enables real-time catalytic amplification within confined volumes, reducing reagent consumption and shortening assay time [89]. These features make microfluidic–nanozyme systems particularly suitable for POC applications, where portability and automation are essential [90]. Integrating nanozymes into microfluidic architectures has been shown to enhance sensitivity whilst supporting scalable, clinically oriented device development [91].

Compared to traditional biosensor assays that often rely on nucleic acid amplification methods such as PCR, which increase workflow complexity and extend turnaround time, amplification-free detection strategies enabled by nanozyme catalysis simplify these workflows whilst maintaining high analytical sensitivity [92]. Notably, nanozyme-assisted CRISPR/Cas platforms where Fe_3_O_4_–Au nanozymes directly catalyse substrate reactions enable rapid nucleic acid detection without enzymatic amplification [93]. This approach streamlines assay design, reduces operational costs, minimizes error rates, and improves compatibility with POC devices [94]. Hence, amplification-free nanozyme biosensors represent a significant shift toward simplified, clinically viable cancer diagnostics.

**Figure 4:**
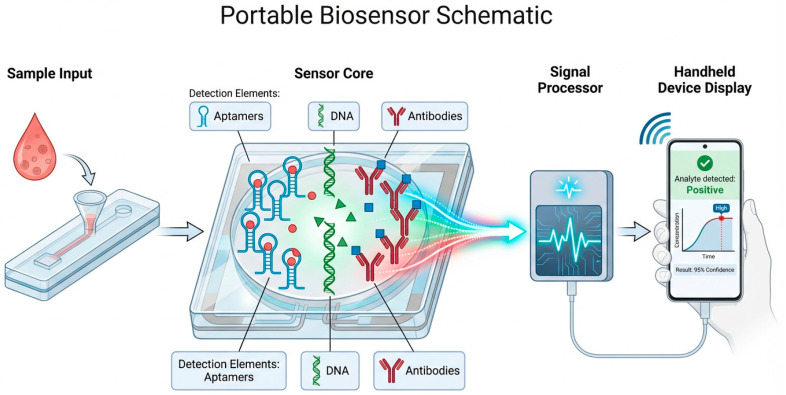
Portable biosensor system integrating sample input, molecular recognition, signal transduction, and real-time digital readout. This schematic illustrates the operational workflow and functional components of a portable biosensor designed for point-of-care cancer diagnostics. (1) Sample Input. A small volume of biological fluid (e.g., whole blood) is introduced into a disposable cartridge. The droplet symbol represents minimally invasive sampling, whilst the cartridge icon denotes a self-contained microfluidic chamber that guides the sample toward the sensing interface. This unit ensures controlled sample delivery, minimizes contamination, and enables rapid onsite testing. (2) Sensor Core with Surface-Functionalized Detection Elements. The central sensing region contains immobilized biorecognition molecules: aptamers, DNA probes, and antibodies, each represented by distinct labels. These elements selectively bind target analytes such as circulating tumour markers, nucleic acids, or proteins. Binding events modulate the physicochemical properties of the sensor surface, triggering a measurable signal. The geometric shapes adjacent to the recognition elements represent analytes interacting with the functionalized surface. This unit performs the critical molecular recognition step that determines assay specificity and sensitivity. (3) Signal Processor. The electronic module receives the raw signal generated by the sensor core, such as fluorescence modulation, electrochemical current changes, or impedance shifts, and converts it into a digital waveform. The waveform graphic symbolizes signal conditioning, amplification, noise filtering, and feature extraction. This processing step ensures that subtle biomolecular interactions are translated into robust, quantifiable outputs suitable for clinical interpretation. (4) Handheld Device Display. The processed signal is transmitted to a portable device (e.g., smartphone or dedicated reader) for real-time visualization. The display panel shows an example output: “Analyte detected: Positive,” accompanied by a concentration versus time plot and a confidence metric (“95% Confidence”). These elements represent quantitative reporting, trend analysis, and decision support features. This unit enables immediate interpretation by clinicians or end users, supporting decentralized diagnostics and rapid clinical decision making. Overall relevance to biosensing applications: together, these components illustrate how portable biosensors integrate molecular recognition, nanozyme-enhanced signal modulation, and digital analytics into a compact, user-friendly platform. The workflow supports point-of-care cancer biomarker detection, enabling rapid, sensitive, and accessible diagnostics outside centralized laboratories. The modular design also facilitates multiplexing, smartphone-based data management, and future integration with AI-assisted interpretation.

Consistent with this, the Figure 4 schematic illustrates how portable biosensor platforms integrate sample handling, molecular recognition, and real-time signal processing. The figure depicts a compact system in which a blood sample is introduced into the device, passes through a sensor core functionalized with aptamers, DNA, and antibodies, and generates a measurable signal upon analyte interaction. This signal is processed electronically and transmitted to a handheld display for immediate interpretation, demonstrating how portable biosensors translate molecular recognition events into clinically actionable readouts.

## 6. Translational and Clinical Perspectives

Despite substantial progress in laboratory research, the clinical translation of nanozyme-enabled biosensors continues to face significant challenges. Validation across diverse patient cohorts remains essential, as diagnostic performance must be demonstrated under real-world conditions to ensure reliability and reproducibility [95]. Regulatory approval also represents a major bottleneck, requiring rigorous assay standardization, robust quality-assurance frameworks, and long-term stability testing to meet international diagnostic compliance standards [96].

Furthermore, scalability of nanozyme synthesis and device manufacturing presents an additional hurdle, as cost-effective production is necessary for widespread adoption in healthcare systems [97]. Without comprehensive validation and regulatory clearance, the transition of nanozyme technologies from bench to bedside will remain constrained.

Nevertheless, nanozyme-enabled biosensors are well positioned to support POC diagnostics, offering rapid, portable, and user-friendly platforms for cancer detection [98]. Integration with smartphones and wearable devices enables decentralized testing, reducing dependence on centralized laboratories and improving access in resource-limited settings [99,100].

By integrating biosensors with mobile technologies, these systems empower both patients and clinicians through improved accessibility, rapid data acquisition, and enhanced clinical decision making in resource-limited settings. These opportunities align with global healthcare priorities to expand early detection and personalized monitoring, improving patient outcomes.

Thus, by linking biosensors with mobile technologies, these platforms empower both patients and clinicians, through improved accessibility, to rapidly acquire data, and enable enhanced clinical decision-making in settings where laboratory infrastructure is limited. Such capabilities align with global healthcare priorities focused on early detection, personalized monitoring, and improved patient outcomes [100].

Extending beyond proof-of-concept for POC applications, nanozyme technologies, including biosensors and therapeutic nanoplatforms, are moving towards personalized medicine, enabling patient stratification based on biomarker profiles for tailored therapeutic interventions in cancer and other diseases [101]. Multiplexed detection of proteins, nucleic acids, and circulating tumour cells provide comprehensive insights into tumour heterogeneity and clonal evolution [102].

A recent nanotheranostics review (2025) highlighted the convergence of diagnostic and therapeutic functions, demonstrating that nanozyme platforms can simultaneously detect biomarkers and deliver targeted interventions [103]. These translational perspectives, together with the expanding diagnostic potential of nanozyme-enabled biosensors, outline a rapidly evolving landscape in which the integration of laboratory innovation into clinical practice is becoming increasingly achievable.

**Figure 5:**
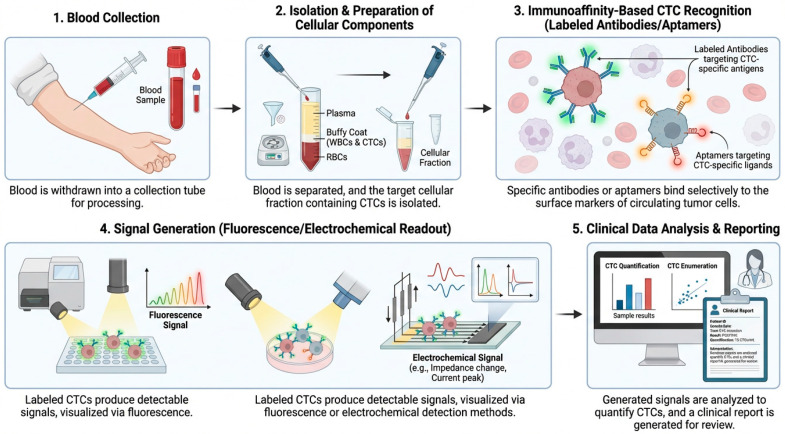
Workflow for CTC detection. Stepwise schematic illustrating the detection of circulating tumour cells (CTCs) from whole blood. The process includes blood collection, isolation and preparation of cellular components, immunoaffinity-based CTC recognition using labelled antibodies or aptamers, signal generation via fluorescence or electrochemical readout, and final data analysis for clinical reporting.

The schematic workflow in Figure 5 illustrates the stepwise process used for CTC detection from whole blood. The diagram outlines each stage from blood collection and cellular preparation to immunoaffinity-based recognition using labelled antibodies or aptamers, followed by signal generation through fluorescence or electrochemical readouts and final data interpretation for clinical reporting.

## 7. Discussion

Nanozyme-enabled biosensing increasingly relies on catalytic systems that combine enzyme-mimetic activity with robust structural design, engineered surface chemistry, and compatibility with miniaturized analytical platforms [104]. Across peroxidase-, oxidase-, and catalase-like nanozymes, signal amplification is governed primarily by defect-engineered active sites and controlled electron transfer pathways, rather than by particle composition itself [105,106]. This mechanistic perspective provides a more predictive framework for understanding biosensor performance and guides rational design strategies for next-generation diagnostic platforms [107].

### 7.1. Mechanistic Determinants of Catalytic Efficiency

The catalytic pathways illustrated in Figure 1 depict how nanozyme activity is fundamentally governed by surface defect density, oxygen-vacancy distribution, and redox-active metal centres [108]. Peroxidase-like nanozymes, particularly Fe_3_O_4_–Au composites, rely on vacancy-mediated adsorption of H_2_O_2_ and rapid electron shuttling between Fe^2+^/Fe^3+^ sites to generate hydroxyl radicals and high-valent metal–oxo intermediates that drive chromogenic oxidation [109]. These processes are highly sensitive to the density of coordinatively unsaturated metal atoms and the electronic coupling between the iron oxide core and gold shell, explaining why engineered Fe_3_O_4_–Au systems frequently achieve catalytic efficiencies comparable to or exceeding horseradish peroxidase [110].

Oxidase-like nanozymes operate through a distinct mechanism in which direct O_2_ activation is enabled by mixed-valence metal centres and defect-rich lattice edges [111]. Cerium oxide and manganese oxide nanozymes exemplify this behaviour, where Ce^3+^/Ce^4+^ redox cycling stabilizes partially reduced oxygen species such as O_2_•^−^ and ^1^O_2_, enabling substrate oxidation without exogenous H_2_O_2_ [112]. This eliminates reagent-dependent steps and simplifies assay workflows, a key advantage for point-of-care deployment [113].

Catalase-like nanozymes, including Pt-based sheets and MnO_2_ nanosheets, catalyse H_2_O_2_ decomposition through two-step redox cycling at high-energy active sites [114]. Lattice strain and engineered surface defects modulate adsorption energies and stabilize reaction intermediates, accelerating turnover and preventing the accumulation of harmful ROS [115]. These mechanistic distinctions highlight how nanozyme classes differ not only in catalytic mode but also in their suitability for specific biosensing contexts [116].

### 7.2. Comparative Performance Across Nanozyme Classes

Table 1, Table 2 and Table 3 collectively demonstrate that nanozyme performance is strongly influenced by active site architecture, defect chemistry, and hybrid material integration [117]. Single-atom nanozymes (SANs) exhibit exceptional catalytic precision due to atomically dispersed M–N_4_ or M–N_6_ coordination environments, which lower activation barriers and enhance electron transfer kinetics [118]. The high turnover rates and stability under harsh conditions make SANs promising candidates for oxidative stress biomarkers and tumour microenvironment sensing [119].

MOF-based nanozymes leverage ordered porosity, high surface area, and tunable metal–ligand coordination to facilitate substrate preconcentration and controlled redox behaviour. Their modularity supports multiplexed detection of nucleic acids, proteins, and metabolites, although their clinical readiness remains limited by structural fragility and synthesis complexity [120].

Cascade nanozyme systems, which integrate sequential catalytic steps (e.g., oxidase–peroxidase or SOD–catalase), offer superior signal amplification and dynamic range by mimicking natural metabolic pathways. The spatially organized active sites enhance intermediate channelling and reduce side reactions, enabling robust performance in complex physiological matrices [121]. The recent advances summarized in Table 3 illustrate how hybrid cascade architectures can outperform single-enzyme mimics in both sensitivity and functional integration [122].

### 7.3. Integration with Biosensor Platforms and Device Miniaturization

The mechanistic advantages of nanozymes translate directly into improved performance across electrochemical, optical, and microfluidic biosensors [123]. Electrochemical platforms benefit from enhanced electron transfer kinetics and catalytic amplification at defect-rich nanozyme interfaces, enabling ultra-low detection limits for proteins, nucleic acids, and tumour metabolites [124]. Optical biosensors, including fluorescence and colorimetric systems, leverage nanozyme-driven ROS generation and substrate oxidation to produce strong, quantifiable signals with high reproducibility [125].

**Figure 6:**
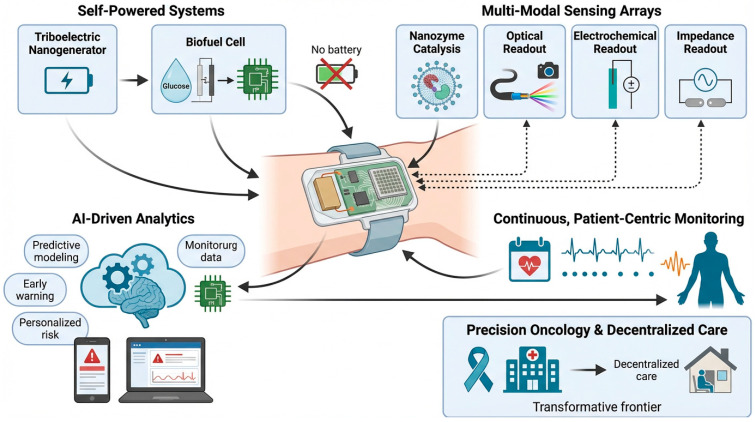
Schematic overview of an integrated wearable biosensing framework illustrating how self-powered modules (triboelectric nanogenerators and biofuel cells) drive continuous operation without external batteries; multimodal sensing arrays (nanozyme-based catalysis, optical, electrochemical, and impedance readouts) enable comprehensive biochemical and physiological monitoring; and AI-driven analytics support predictive modelling, early warning alerts, and personalized risk assessment. The system demonstrates a pathway toward continuous patient-centric monitoring and precision oncology within decentralized care environments.

Microfluidic integration (Figure 6) further strengthens translational potential by enabling real-time catalytic amplification within confined volumes, reducing reagent consumption, and supporting automated workflows [126]. When combined with self-powered systems and AI-driven analytics, nanozyme-based microfluidic devices offer a pathway toward decentralized cancer diagnostics, continuous monitoring, and precision oncology [127].

### 7.4. Translation Barriers: Variability, Scalability, and Regulatory Pathways

Despite these advances, several barriers must be addressed before nanozyme enabled biosensors can achieve clinical adoption. Batch-to-batch variability remains a significant challenge, as small deviations in defect density, surface ligand distribution, or metal coordination environments can produce substantial fluctuations in catalytic activity [128,129]. This variability complicates assay standardization and undermines reproducibility across laboratories.

Scalability is another major limitation. Many high performing nanozymes are synthesized through laboratory scale routes that do not translate well to industrial manufacturing, where maintaining uniform defect structures and catalytic stability is difficult [130]. Additionally, regulatory frameworks for nanozyme-based diagnostics are still emerging, with stringent requirements for physicochemical characterization, long-term biocompatibility, environmental safety, and quality control [131]. Addressing these challenges will require coordinated efforts in materials engineering, process optimization, and regulatory science [132].

**Table 3:** Recent advances in single-atom, MOF-based, and cascade nanozyme systems for cancer-relevant biosensing (last 3–5 years).

Nanozyme Class	Representative System & Catalytic Activity	Mechanistic Features (Surface/Defect Level)	Biosensing/Biomedical Relevance	Relative Performance vs. Natural Enzymes	References
Single-atom nanozymes	Atomically dispersed Fe, Rh, V, or Fe–Cu centres coordinated in N-doped carbon (e.g., Fe–N_4_, Rh–N_4_, VN_4_) with peroxidase-, catalase-, and SOD-like activities	Isolated metal sites with well-defined coordination (M–N_4_, M–N_6_) lower activation barriers, stabilize high-valent metal–oxo intermediates, and enable highly efficient electron transfer; defect engineering and local electronic structure tuning control activity and selectivity	High-affinity ROS modulation, peroxidase- and catalase-like catalysis, antioxidative activity; adaptable to electrochemical and optical biosensing of oxidative-stress biomarkers and tumour microenvironment parameters	Catalytic affinities and turnover can exceed natural peroxidase and catalase (e.g., RhN_4_ and VN_4_ outperforming HRP and catalase), with superior stability under harsh conditions	[133,134,135]
MOF-based nanozymes	Transition-metal MOFs (Fe-, Co-, Cu-based) exhibiting peroxidase-, oxidase-, or multi-enzyme-like activities	High surface area and ordered porosity facilitate substrate diffusion and preconcentration; metal nodes act as catalytic centres; organic linkers and defect sites modulate redox properties and electron transport; structural tunability optimizes active-site density and microenvironment	Colorimetric and electrochemical detection of small molecules, ROS, and biomacromolecules; integration into hybrid platforms (e.g., MOF–Prussian blue, MOF–graphene) for enhanced signal amplification and multiplexed cancer biomarker detection	Comparable or higher stability than natural enzymes; catalytic efficiency enhanced via defect engineering, metal-node selection, and pore-environment control, enabling sensitive biosensing at pM–nM levels	[136,137]
Cascade nanozyme systems	Hybrid constructs combining sequential activities (oxidase–peroxidase, SOD–catalase, multi-step ROS modulation)	Spatially organized catalytic sites enable stepwise conversion of substrates (O_2_ → ROS → oxidized chromogen); defect-rich interfaces and controlled electron transfer pathways coordinate multiple reactions; nano-confinement enhances intermediate channelling and reduces side reactions	Signal amplification via tandem reactions, improved dynamic range, and closer mimicry of natural metabolic/redox cascades; relevant to tumour microenvironment sensing, multi-analyte detection, and theranostic platforms	Although individual step kinetics may be lower than optimized single enzymes, overall cascade performance yields superior amplification, robustness, and functional integration under physiological conditions	[138,139]

## 8. Outlook

The mechanistic insights synthesized in this review highlight the importance of defect engineering, electron transfer optimization, and hybrid material design in advancing nanozyme-enabled biosensing. Continued progress in single-atom catalysis, MOF-hybrid architectures, and cascade nanozyme systems will likely yield platforms with higher catalytic precision, improved robustness, and enhanced multiplexing capability. Integration with microfluidics, wearable systems, and AI-assisted analytics (Figure 2, Figure 3, Figure 4, Figure 5 and Figure 6) positions nanozyme-based biosensors as strong candidates for next-generation cancer diagnostics.

However, realizing their full clinical potential will depend on overcoming variability, achieving scalable manufacturing, and establishing regulatory pathways that ensure safety and reproducibility. With these challenges addressed, nanozyme-enabled biosensors could play a transformative role in early cancer detection, real-time monitoring, and decentralized precision oncology.

## 9. Conclusions

Nanozyme-enabled biosensors are entering a decisive translational phase, with clear progress across POC platforms, portable diagnostic systems, and multiplexed analytical workflows. These advances directly support global healthcare priorities by decentralizing diagnostic capacity, improving accessibility, and enabling more individualized clinical decision making. Emerging capabilities in circulating tumour cell detection, biomarker profiling, and amplification-free portable assays demonstrate how nanozyme technologies can effectively bridge laboratory innovation with clinical practice, accelerating the shift toward precision oncology.

This review establishes nanozyme-enabled biosensors as a coherent roadmap for next-generation diagnostic technologies. By integrating catalytic principles, nanomaterial engineering, translational workflow design, and emerging directions such as AI-driven analytics and multi-omics convergence, we outline a comprehensive framework for advancing the field. The long-term impact of nanozyme platforms will depend on sustained progress in materials optimization, scalable manufacturing, ethical deployment, and rigorous regulatory validation.

## Figures and Tables

**Table 1:** Comparison of nanozyme types, catalytic activities, and cancer biomarker uses.

Nanozyme	Catalytic Activity	Application	Reference
Fe_3_O_4_ nanoparticles	Peroxidase-like	Colorimetric detection of H_2_O_2_ and glucose in tumour microenvironment	[43]
Cerium oxide (CeO_2_)	Oxidase-like	ROS scavenging and tumour imaging	[44]
Carbon nanodots	Peroxidase-like	Electrochemical detection of PSA	[45]
MnO_2_ nanosheets	Catalase-like	Oxygen generation for hypoxia relief in tumours	[46]
Gold nanoparticles (AuNPs)	Glucose oxidase mimic	Fluorescent detection of glucose and lactate	[47]
Copper-based nanozymes	Superoxide dismutase-like	ROS modulation and biosensing of cancer-related oxidative stress	[48]
Composite bimetallic nanozymes (Fe-based)	Peroxidase mimicking	Chemodynamic therapy (CDT) and multiplexed biomarker detection	[49]

**Table 2:** Expanded comparison of nanozyme classes.

Nanozyme Class	Catalytic Activity	Detection Modality	Sensitivity (LOD)	Cancer Biomarker Application	Clinical Readiness	Reference
Metal–organic frameworks (MOFs)	Peroxidase/oxidase-like	Electrochemical, colorimetric	pM–nM range	Multiplexed detection of miRNA, PSA, and CTCs	Preclinical	[50]
Prussian blue nanoparticles	Peroxidase/catalase/SOD-like	Colorimetric, electrochemical	nM range	Detection of alkaline phosphatase, ROS, and tumour metabolites	Early clinical	[51]
Graphene-based nanozymes	Peroxidase/oxidase-like	Electrochemical, fluorescence	fM–pM range	PSA, miRNA, and exosome detection	Preclinical	[52]
Carbon nanotube composites	Peroxidase-like	Electrochemical	pM range	Multiplexed biosensing of tumour markers	Preclinical	[53]
MOF–Prussian blue hybrids	Dual peroxidase/catalase	Electrochemical, colorimetric	nM range	ROS modulation and biomarker detection	Preclinical	[54]
Graphene quantum dots	Oxidase-like	Fluorescence, FRET	fM range	miRNA and protein biomarker detection	Preclinical	[55]

Key notes: MOFs: Highly porous, tunable structures allow multiplexed biomarker detection but remain at preclinical proof-of-concept stage. Prussian blue: Biocompatible and stable, already evaluated in early clinical diagnostics for ROS and enzyme-linked biomarkers. Graphene-based nanozymes: Exceptional sensitivity (down to femtomolar levels) due to high conductivity and surface area, but clinical translation is still limited. Hybrid systems (MOF–PB, CNT composites): Offer synergistic catalytic activities, promising for multiplexed detection, though still in preclinical validation.

## Data Availability

No new data were created or analyzed in this study.

## Abbreviations

The following abbreviations are used in this manuscript:

AI	Artificial Intelligence
AuNPs	Gold Nanoparticles
CA15-3	Cancer Antigen 15-3
CEA	Carcinoembryonic Antigen
CDT	Chemodynamic Therapy
CeO_2_	Cerium Oxide
CTC(s)	Circulating Tumour Cell(s)
ctDNA	Circulating Tumour DNA
CNTs	Carbon Nanotubes
DNA	Deoxyribonucleic Acid
ELISA	Enzyme-Linked Immunosorbent Assay
FRET	Förster Resonance Energy Transfer
Fe_3_O_4_-Au	Iron Oxide–Gold Composite
GO	Graphene Oxide
GQD	Graphene Quantum Dot
HER2	Human Epidermal Growth Factor Receptor 2
HRP	Horseradish Peroxidase
LOD	Limit of Detection
miRNA	MicroRNA
MOF(s)	Metal–Organic Framework(s)
MSNs	Mesoporous Silica Nanoparticles
MnO_2_	Manganese Dioxide
MUC1	Mucin-1
PANI	Polyaniline
PB	Prussian Blue
POC	Point-of-Care
PSA	Prostate-Specific Antigen
QDs	Quantum Dots
QD@MSN-EPI-Au-PEG-Apt	QDS-Quantum Dots; MSN-Mesoporous Silica Nanoparticle Shell; EPI-Epirubicin; Au-Gold; PEG-Polyethylene Glycol; Apt-Aptamer
RBCs	Red Blood Cells
ROS	Reactive Oxygen Species
SNP	Silica Nanoparticle
SOD	Superoxide Dismutase
SANs	Single-Atom Nanozymes
TEM	Transmission Electron Microscopy
WBCs	White Blood Cells
ZIF-8	Zeolitic Imidazolate Framework-8
